# Plasma carotenoids are associated with socioeconomic status in an urban Indigenous population: an observational study

**DOI:** 10.1186/1471-2458-11-76

**Published:** 2011-02-02

**Authors:** Allison Hodge, Joan Cunningham, Louise Maple-Brown, Terry Dunbar, Kerin O'Dea

**Affiliations:** 1 Cancer Epidemiology Centre, Cancer Council Victoria, Melbourne, Australia; 2 Menzies School of Health Research, Institute of Advanced Studies, Charles Darwin University, Darwin, Australia; 3 Division of Medicine, Royal Darwin Hospital, Darwin, Australia; 4 Faculty of Education, Health and Science and Graduate School of Health Practices. Charles Darwin University, Australia; 5 Sansom Institute for Health Research, University of South Australia, Australia

## Abstract

**Background:**

Indigenous Australians experience poorer health than other Australians. Poor diet may contribute to this, and be related to their generally lower socioeconomic status (SES). Even within Indigenous populations, SES may be important. Our aim was to identify factors associated with plasma carotenoids as a marker of fruit and vegetable intake among urban dwelling Indigenous Australians, with a particular focus on SES.

**Methods:**

Cross sectional study in urban dwelling Indigenous Australians participating in the DRUID (Darwin Region Urban Indigenous Diabetes) Study. An SES score, based on education, employment, household size, home ownership and income was computed and plasma carotenoids measured by high performance liquid chromatography in 897 men and women aged 15 - 81 years (mean 36, standard deviation 15). Linear regression analysis was used to determine the relationship between SES and plasma carotenoids, adjusting for demographic, health and lifestyle variables, including frequency of intakes of food groups (fruit, vegetables, takeaway foods, snacks and fruit/vegetable juice).

**Results:**

SES was positively associated with plasma concentrations of lutein/zeaxanthin (p trend <0.001), lycopene (p trend = 0.001), α- and ß-carotene (p trend = 0.019 and 0.026 respectively), after adjusting for age, sex, glucose tolerance status, smoking, alcohol use, hypercholesterolemia, dyslipidemia, self-reported health, waist to hip ratio and body mass index. These associations remained after adjustment for self-reported frequency of intake of fruit, vegetables, takeaway foods and fruit juice, which all showed some association with plasma carotenoids. Even in the highest SES quintile, concentrations of all carotenoids (except lycopene) were lower than the mean concentrations in a non-Indigenous population.

**Conclusions:**

Even within urban Indigenous Australians, higher SES was associated with higher concentrations of plasma carotenoids. Low plasma carotenoids have been linked with poor health outcomes; increasing accessibility of fruit and vegetables, as well as reducing smoking rates could increase concentrations and otherwise improve health, but our results suggest there may be additional factors contributing to lower carotenoid concentrations in Indigenous Australians.

## Background

Indigenous Australians are at increased risk of cardiovascular disease, type 2 diabetes and renal disease compared with other Australians [1,2]. While the reasons for this are complex, poor diet is likely to be important [2-4]. Fresh fruit and vegetables are often in limited supply in stores in remote Aboriginal communities [5], and previous studies of Aborigines from one such community have demonstrated low concentrations of plasma carotenoids [3,4], biomarkers of fruit and vegetable intake.

Low plasma carotenoid concentrations were found to be associated with urinary albumin excretion in a study of Australian Aborigines from north west Australia, indicating a potential association with vascular renal disease [3]. An inverse association has also been reported between plasma carotenoids and C-reactive protein (CRP), a marker of inflammation [6]. There is evidence that plasma or serum carotenoid concentrations are inversely associated with cardiovascular disease (CVD) risk [7]. This may be due to the antioxidant actions of carotenoids themselves [7], or because carotenoids are a marker of higher intakes of fruit and vegetables [8] and overall diet quality [9].

In remote Indigenous communities, limited availability and high cost of fresh fruit and vegetables are a major contributor to poor quality dietary intake [10]. In urban areas, despite improved availability, low socioeconomic status (SES) may still be an important determinant of the purchase and consumption of fresh fruit and vegetables, as has been demonstrated in other studies internationally [11]. If improving overall diet quality, as indicated by plasma carotenoid concentrations, improves health in Indigenous Australians, better understanding of how SES relates to this will be important.

The primary aim of this study was to evaluate the relationship between SES and plasma carotenoids in an urban Indigenous population, and the extent to which this relationship is mediated by self-reported intake of fruit and vegetables.

## Methods

Data were collected in 2003-2005 as part of the DRUID (Darwin Region Urban Indigenous Diabetes) Study, which has been described in detail elsewhere [12]. Briefly, eligible participants were volunteers aged 15 years or more who identified themselves as Aboriginal and/or Torres Strait Islander, had lived within a defined geographic region in and around the city of Darwin for at least 6 months, and did not live in an institutional dwelling. The area, known as the Yilli Rreung Aboriginal and Torres Strait Islander Commission Region, was estimated to have a total Indigenous population around 12,000 in the 2001 Census. In the absence of a sampling frame it was not possible to determine a true response rate, but it is estimated that about 14% of eligible people participated. The recruitment strategy did not systematically contact all the estimated 7051 eligible people, rather a variety of strategies using existing networks were used to recruit as many people as possible. While it is not possible to compare responders with non-responders, comparison with census data and data from the Northern Territory Department of Health and Community Services suggested that the participants were more likely to be female and participating females were older than the target population [12]. However, overall participants were similar in terms of age, place of residence, Indigenous group and household income in comparison to the local population in the 2001 Census [13].

### Participants

A total of 1,004 eligible people participated and provided at least one measurement, of these, 107 people did not have carotenoid data, leaving 280 men and 617 women with a mean age of 36.6(standard deviation (SD) 14.6) years, who were included in these analyses.

### Blood sampling

For participants who had fasted for at least 10 hours, fasting blood samples were collected. Up to 13 ml of blood was collected in tubes appropriate for each assay. Blood samples were stored on ice until centrifuged at 3000 RPM for 15 minutes. Where possible this was completed within 1 hour of collection.

### Measurements

Socioeconomic status was represented in this analysis by self-reported information on education, employment, household size (as a proxy for overcrowding), home ownership and income [14]. Weekly household income was collected using eight categories: $1-79 per week; $80-199; $200-399; $400-599; $600-799; $800-1,499; $1,500 or more; and 'Don't know'. Household members were counted as adults aged 15 and over, or children. Gross weekly household equivalised income was calculated using the mid-point of each income range, or $1600 for the top level, and dividing by sum of the weights for all household members, according to the "OECD-modified scale". This scale, first proposed by Hagenaars [15] assigns a value of 1 to the household head, 0.5 to each additional adult member and 0.3 to each child under 14 years. This implies that for an additional adult a household needs an extra 50% of resources above that used by the first adult to maintain the same standard of living; or 30% more for each child. The results were categorised as $1-199, $200-499, and $500+. Educational qualifications were categorised as None, Year 10/Year 12, Trade certificate/apprenticeship/diploma, and University degree or higher. These two latter groups were combined as post school qualifications in Table 1. Employment status was represented by whether or not the participant was in full-time work. The number of people in the household was categorised as 1-2, 3-4, and 5+. Home ownership was categorised as living in a dwelling that was owned or being purchased by its occupants, or renting/other tenure.

**Table 1:** Characteristics of participants by gender

Variable	Men n = 280	Women n = 617
***Numbers and (proportions)***		

*Ethnic origin*		
Aboriginal	233 (79.6)	531 (86.1)
Torres Strait Islander	18 (6.4)	30 (4.9)
Both	39 (13.9)	56 (9.1)

Current smoker	113 (40.4)	247 (40.0)

Alcohol consumer	198 (70.7)	375 (60.8)

*Glucose tolerance*		
Diabetic	42 (15.0)	119 (19.3)
Abnormal glucose tolerance^a^	36 (12.9)	83 (13.4)
Normal glucose tolerance	180 (64.3)	364 (59.0)
Unclassified	22 (7.9)	51 (8.3)

Hypertension	52 (18.6)	114 (18.5)

Hypercholesterolaemia	117 (41.8)	195 (31.6)

Dyslipidaemia^b^	141 (50.4)	233 (37.8)

Takeaway 2+/wk	105 (37.5)	153 (24.8)

Snacks 2+/wk	107 (38.2)	228 (37.0)

4+ veg serves/day^c^	25 (8.9)	56 (9.1)

2+ fruit serves/d	88 (31.4)	219 (35.5)

1+ Fruit/veg juice serves/d	41 (14.6)	98 (15.9)

Sufficient fruit & veg^d^	17 (6.1)	41 (6.6)

Household equivalised income ^e ^($/week)		
1-199	62 (22.1)	134 (21.7)
200-499	77 (27.5)	171 (27.7)
500	65 (23.2)	133 (21.6)
Missing	76 (27.1)	179 (29.0)

In full time work	111 (36.6)	243 (39.4)

Post school qualification	82 (29.3)	189 (30.6)

Home is owned or being purchased	105 (37.5)	236 (38.2)

5+ in household	73 (26.1)	194 (31.4)

Manage OK on income	146 (52.1)	284 (46.0)

Rate health as good or excellent	194 (69.3)	422 (68.4)

***Medians (25***^***th***^***-75***^***th***^***%iles)***		

Age (yrs)	35 (22-46)	37 (25-48)

Waist circumference (cm)	95 (85-105)	93 (81-104)

Waist to hip ratio	0.94 (0.89-1.00)	0.87 (0.81-0.93)

Body mass index (kg/m^2^)	27.0 (23.5-30.9)	27.8 (23.5-32.9)

^a ^Impaired glucose tolerance and impaired fasting glucose combined.

^b ^Triglycerides ≥ 1.7 or HDL <0.9 (male), <1.0 (female)

^c ^The response categories for vegetable frequency mean we cannot look at 5+ serves per day as per current recommendations.

^d ^Defined as at least four veg and two fruit/day see point b above.

^e ^**"OECD-modified scale"**. After having used the "old OECD scale" in the 1980s and the earlier 1990s, the Statistical Office of the European Union (EUROSTAT) adopted in the late 1990s the so-called "OECD-modified equivalence scale". This scale, first proposed by Haagenars *et al. *(1994), assigns a value of 1 to the household head, of 0.5 to each additional adult member and of 0.3 to each child.

A 2-hour oral glucose tolerance test was administered to all consenting participants except those using medication for previously diagnosed diabetes, and those who were pregnant. Glucose was measured in plasma and diabetes was defined as: 1) fasting plasma glucose ≥ 7.0 mmol/L; 2) 2-hour post-glucose load plasma glucose ≥ 11.1 mmol/L; or 3) previously diagnosed as having diabetes and currently taking tablets and/or insulin for diabetes. Impaired glucose tolerance (IGT) was defined as fasting glucose <7.0 mmol/L and 2-hour glucose ≥ 7.8 and <11.1 mmol/L; impaired fasting glucose (IFG) as fasting glucose ≥ 6.1 and <7.0 mmol/L, and 2-hour glucose <7.8 mmol/L. Normal glucose tolerance (NGT) included all those with fasting glucose <6.1 mmol/l and 2-hour glucose <7.8 mmol/l.

Total cholesterol, triglycerides, and HDL cholesterol were measured in fasting serum as previously described [12]. Blood samples were frozen after processing and shipped to the Clinical Trials Laboratory at Flinders Medical Centre (Bedford Park, South Australia) for analysis. Dyslipidemia was defined using the World Health Organization criteria: fasting HDL cholesterol <0.9 mmol/L (men), <1.0 mmol/L (women); or fasting triglycerides ≥ 1.7 mmol/L [16]. Hypercholesterolaemia was defined as total cholesterol >5.5 mmol/l or self-reported use of cholesterol lowering medication.

The extraction and analysis of individual carotenoids (lycopene, lutein, zeaxanthin, alpha- and beta-carotene), retinol and tocopherols (alpha- and gamma-tocopherol) was based on the method of Su et al [17]. Briefly, 200 μl of plasma was extracted twice with 1 ml of Hexane containing 0.01% BHT. For quantification, an internal standard of Echinenone 0.167 μg/ml was added to all samples prior to the extraction. The extract was dried under nitrogen at room temperature, then reconstituted in 100 μl of mixture CHCl_3_:MeOH:CH_3_CN (30:35:35). Fifty μl was injected into the high performance liquid chromatograph (HPLC) (Shimadzu HPLC machine equipped with an SPD-M20A PDA Detector and a NovoPak C^18 ^column) with absorbance detection at 292 nm for the tocopherols, 325 nm for retinol and 450 nm for the carotenoids. The carotenoids, retinol and tocopherols were eluted from the column using a mobile phase of 0.0125% ammonium acetate in MeOH (A), 100% CHCl_3 _(B) and CH_3_CN with 0.1% triethylamine (C) in three linear gradient steps: from 0 to 5 min, A 50%, C decreased from 50% to 44% and B increased to 6%; from 5 to 16 min, A increased to 55%, C decreased from 44% to 30% and B increased from 6% to 15%. Wash with A:C 50/50 mixture for 3 mins. Retinol and tocopherols in plasma are not good biomarkers of diet [18] and are not considered further.

Body weight was recorded to the nearest 0.1 kilogram using a digital scale weighing up to 200 kg ( Model 767, Seca Deutschland, Hamburg, Germany), and height to the nearest 0.1 cm using a portable stadiometer (Model PE87, Mentone Educational Centre, Moorabbin, Victoria, Australia). Waist and hip circumferences were measured to the nearest 0.1 cm using a 2-metre non-stretch fiberglass tape. The waist was defined as the mid-point between the iliac crest and the costal margin, and the hips as the widest circumference over the buttocks and below the iliac crest. Abdominal obesity was defined as a waist/hip ratio (WHR) of >0.9 for men and >0.8 for women or waist circumference >102 cm for men or >88 cm for women. Participants were classified as underweight if body mass index (BMI) <20, normal weight if BMI ≥ 20 and ≤25, overweight if BMI >25 and ≤ 30, and obese if BMI >30 kg/m^2 ^for both men and women.

Sitting blood pressure was measured using a Welch Allyn Spot Vital Signs monitor (Welch Allyn Medical products, Skaneateles Falls, USA). Hypertension was defined as systolic blood pressure ≥ 140 mmHg and/or diastolic blood pressure ≥ 90 mm Hg and/or current anti-hypertensive medication [16].

Current tobacco smokers and alcohol consumers were identified by self-report using a simple yes/no response. Self-rated health (excellent, very good, good, fair, poor) was reported. The dietary data was based on questions frequency of consumption of selected food groups. For 'Fruit' and 'Vegetables' consumed per day, response options were 'None' '1 serve or less', '2-3 serves', 4-5 serves', '6 or more serves' or 'Don't know/not sure'. Frequency of consumption of food groups 'Takeaway foods', 'Snacks' and 'Fruit/vegetable juice' was recorded using response options 'Never or rarely', Less than once a week', '1 time per week', '2-3 times per week', '4-6 times per week', 1 time per day' '2 times per day', '3 or more times per day'.

### Ethics approval

The study was approved by the Human Research Ethics Committee of the Northern Territory Department of Health & Community Services and Menzies School of Health Research. It was considered and approved by both the Aboriginal sub-committee, which has absolute right of veto, and by the main committee. The study's governance structure included an Indigenous Steering Group, as well as partnerships with key Indigenous organisations [12]. All participants gave informed consent before undergoing the health examination.

### Statistical methods

Initially, associations between SES variables and plasma carotenoids were evaluated separately to identify those that showed associations (p < 0.05): education, employment, household size, home ownership and income. Subsequently a combined score was developed by including the five variables identified as having univariate associations in factor analysis using the principal factor method [19] and then computing a factor score for each individual based on the loadings for each variable. Due to missing data on one or more of these variables, factor scores could only be computed for 600 of 897 participants. Further analysis revealed that people missing the factor score tended to be similar to people in the middle 20% of the factor score distribution for variables they were not missing, and so they were allocated to this group.

Plasma carotenoids were not normally distributed so medians, 25^th ^and 75^th ^percentiles were used to reflect the distributions. Analysis of variance using natural log transformed carotenoids was used to identify variables associated with plasma carotenoid concentrations. Linear regression models including all variables showing univariate associations with at least one of the carotenoids were then computed. Because younger people could not be properly scored on the SES variable as many were still completing their education, the models were also run excluding people aged less than 20 years, leaving 722 for analysis. The results are presented for non-transformed carotenoid variables so that ß-coefficients are more readily interpreted. Using natural log transformed variables did not materially change the patterns of relationships. Models included: the combined SES score as quintile groupings, age (10 yr age groups), gender, BMI (categorical), WHR-obese (Y/N), hypercholesterolaemia (Y/N), dyslipidaemia (Y/N), glucose tolerance (normal, abnormal (IGT and IFG), diabetes), alcohol consumption (Y/N) and smoking (Y/N), all of which have been associated with carotenoid concentrations and are CVD risk factors. People with missing values were classified separately. A second model was computed for each carotenoid variable including frequency of intakes of vegetables, fruit, takeaway food (tomato sauce and pizzas are a good source of lycopene [20]) and fruit/vegetable juice to assess if these dietary measures mediated any associations between SES and plasma carotenoids. A test for linear trend across SES groups was performed by allocating the median factor score to individuals in each group. An alternative outcome variable was constructed comparing people who had plasma carotenoids in the top quartile for each, against the others. This 'hi carotenoid' variable was tested in logistic regression models with the same variables as above. All analyses were performed using STATA version 10 (Stata Corporation, College Station, TX).

## Results

Table 1 presents characteristics of men and women included in the study. The study population was predominantly of Aboriginal origin, with a small proportion reporting Torres Strait Islander background. Large proportions of both men and women were current smokers and consumers of alcohol. Although the median BMIs for men and women were not in the obese range, both median waist circumference and WHR were in the obese range for women, and WHR was in the obese range for men. Only 6-7% of participants reported a sufficient daily intake of fruit and vegetables, defined as at least 4 vegetable and 2 fruit serves.

**Figure 1:**
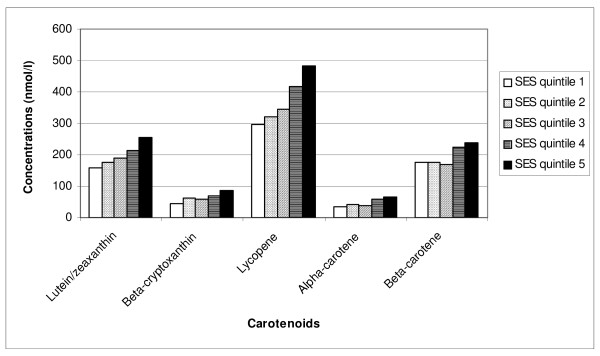
**Mean plasma carotenoid concentrations by quintile of socio-economic status (SES)**.

Significant positive correlations (rho <0.05) were seen between education, employment, home ownership and income, while household size was weakly inversely associated with equivalised income. This suggests that it is appropriate to combine them. Only one SES factor with an Eigen value greater than 1 (1.43) was identified. Positive loadings were seen for home ownership (0.37), educational level (0.45), equivalised income (0.76) and work status (none, part-time, full-time) (0.69). Household size showed a weak inverse loading (-0.17). As shown in Figure 1, unadjusted concentrations of each plasma carotenoid increased across quintiles of SES score (p < 0.001 for all in ANOVA).

In the univariate analysis (data not shown), plasma concentrations of all carotenoids increased with increasing frequency of vegetable or fruit consumption (serves/day); with p < 0.05 for ANOVA in all but lycopene. There were similar trends for consumption of fruit or vegetable juice. Intake of snack food was not associated with plasma carotenoids, but frequency of consumption of takeaway foods (times/week) was inversely associated with all carotenoids except lycopene.

In multivariate models not including dietary intake variables (Table 2), plasma concentrations were higher in the top 20% of the SES score than in the lowest 20% for each carotenoid and tests for linear trend were significant, except for ß-cryptoxanthin. Excluding the people age less than 20 years made very little difference to the associations between carotenoids and SES. For all but lycopene, plasma carotenoids tended to be higher in the older age groups. Hypercholesterolaemia was consistently associated with higher concentrations of carotenoids; conversely, dyslipidaemia (high triglycerides and/or low HDL cholesterol) was associated with lower concentrations of lycopene, α- and ß-carotene. ß-cryptoxanthin, lycopene, and α- carotene were higher in people with normal glucose tolerance (NGT) than those with diabetes. There were no significant associations between WHR and carotenoids. People classified as obese according to BMI tended to have lower carotenoid concentrations than those with a normal BMI, but this only reached significance for lutein/zeaxanthin and ß-carotene. Smokers had lower plasma concentrations of all carotenoids than non-smokers, while alcohol use was associated with higher lycopene and α-carotene than in non-drinkers.

**Table 2:** Beta coefficients and 95% confidence intervals from multivariate models^a ^looking at factors associated with plasma carotenoid concentrations

Variables	Lutein/zeaxanthin (nmol/L)	ß-cryptoxanthin (nmol/L)	Lycopene (nmol/L)	α-carotene (nmol/L)	ß-carotene (nmol/L)
*Age group (yrs)*					
15-24	1.00	1.00	1.00	1.00	1.00
25-34	17.26 (-5.04, 39.55)	-3.92 (-26.67, 18.83)	-2.11 (-46.96, 42.74)	13.55 (319, 23.92)	42.44 (-1.07, 85.95)
35-44	-2.64 (-25.73, 20.46)	4.04 (-19.54, 27.63)	-64.14 (-110.60, -17.69)	16.61 (5.87, 27.36)	79.39 (34.32, 124.46)
45-54	23.62 (-1.31, 48.55)	49.15 (23.71, 74.58)	-45.19 (-95.33, 4.95)	25.23 (13.65, 36.82)	134.24 (85.59, 182.89)
55-64	36.62 (4.67, 68.56)	47.02 (14.42, 79.61)	-125.88 (-190.13, -61.63)	15.65 (0.80, 30.49)	166.36 (104.02, 228.69)
65+	48.33 (5.16, 91.50)	66.47 (22.42, 110.52)	-51.74 (-138.57, 35.09)	24.24 (4.19, 44.30)	164.02 (79.78, 248.27)

Sex					
Male	1.00	1.00	1.00	1.00	1.00
Female	8.26 (-7.48, 23.99)	5.15 (-10.92, 21.23)	-33.15 (-64.79, -1.50)	13.18 (5.87, 20.49)	75.80 (45.09, 106.50)

*SES quintile*					
1	1.00	1.00	1.00	1.00	1.00
2	11.59 (-15.86, 39.04)	4.79 (-23.27, 32.86)	20.66 (-34.56, 73.87)	0.35 (-12.41, 13.10)	28.85 (-24.72, 82.41)
3	30.01 (6.15, 53.87)	1.78 (-22.63, 26.20)	14.82 (-33.17, 62.81)	-3.29 (-14.38, 7.80)	-6.05 (-52.60, 40.51)
4	65.51 (37.18, 93.84)	6.87 (-22.12, 35.87)	47.44 (-9.55, 104.43)	7.44 (-5.75, 20.64)	23.95 (-31.35, 79.24)
5	88.49 (59.51, 117.47)	21.58 (-8.04, 51.20)	105.22 (46.94, 163.51)	17.29 (3.82, 30.75)	86.10 (29.55, 142.65)
p-trend	<0.001	0.202	0.001	0.019	0.026

Poor or fair health	1.00	1.00	1.00	1.00	1.00
Good or better health	8.75 (-9.33, 26.82)	0.83 (-17.63, 19.29)	6.78 (-29.58, 43.14)	9.24 (0.84, 17.64)	34.64 (-0.63, 69.29)

Normal cholesterol	1.00	1.00	1.00	1.00	1.00
Hypercholesterolaemia	40.20 (24.15, 56.25)	19.92 (3.52, 36.31)	91.31 (59.03, 123.59)	12.53 (5.06, 20.00)	76.62 (45.30, 107.94)

No dyslipidaemia	1.00	1.00	1.00	1.00	1.00
Dyslipidaemia	-14.60 (-30.64, 1.43)	-14.12 (-30.48, 2.23)	-65.87 (-98.11,-33.62)	-13.27 (-20.73, -5.82)	-63.38 (-94.67, -32.10)

*Glucose tolerance*					
Normal	1.00	1.00	1.00	1.00	1.00
Abnormal^b^	-16.59 (-39.16, 5.98)	-24.83 (-47.88, -1.77)	-44.21 (-89.62, 1.19)	-17.98 (-28.48, -7.49)	-105.20 (-149.26, -61.15)
Diabetic	1.28 (-21.09, 23.65)	-27.59 (-50.45, -4.73)	-74.27 (-119.26, -29.27)	-22.63 (-33.03, -12.23)	-140.07 (-183.73, 96.41)

Waist to hip ratio					
Non-obese	1.00	1.00	1.00	1.00	1.00
Obese	-3.19 (-23.42, 17.04)	-11.34 (-31.98, 9.30)	-0.28 (-40.97, 40.41)	1.62 (-7.80, 11.04)	-2.75 (-42.23, 36.73)

Body mass index					
Underweight	-11.47 (-38.57, 15.63)	-6.04 (-33.69, 21.61)	4.84 (-49.67, 59.35)	-0.45 (-13.15, 12.25)	-7.03 (-59.91, 45.86)
Normal	1.00	1.00	1.00	1.00	1.00
Overweight	-0.23(-20.56, 20.10)	5.26 (-15.48, 26.00)	29.20 (-11.69, 70.08)	8.03 (-1.42, 17.47)	-12.25 (-51.92, 27.42)
Obese	-23.88 (-45.09, -2.69)	-7.34 (-29.00, 14.31)	-14.13 (-56.79, 28.53)	-4.52 (-14.37, 5.33)	-57.15 (-98.31, -15.76)

Non-smoker	1.00	1.00	1.00	1.00	1.00
Current smoker	-42.53 (-58.39, -26.68)	-52.07(-68.26, -35.88)	-44.87 (-76.77, -12.97)	-17.66 (-25.03, -10.29)	-76.79 (-107.74, -45.84)

Non-alcohol consumer	1.00	1.00	1.00	1.00	1.00
Alcohol consumer	9.69 (-7.29, 26.66)	-4.26 (-21.63, 13.11)	42.14 (7.99, 76.29)	8.17 (0.27, 16.07)	17.44 (-15.69, 50.57)

Adjusted R^2^	0.15	0.10	0.15	0.15	0.21

^a ^The models included only those variables shown in the table.

^b ^Impaired glucose tolerance and impaired fasting glucose combined.

With the addition of dietary data to the model, most of these associations were retained: β-coefficient for quintile 5 vs quintile 1 and p trend for lutein/zeaxanthin (84.53, 55.47-113.58; <0.001), β-cryptoxanthin (20.74, -8.56-50.04; 0.306), lycopene (101.63, 42.48-160.78; 0.003), α-carotene (16.68, 3.13-30.23; 0.035) and β-carotene (90.69, 33.78-147.60; 0.024). Takeaway intake was inversely associated with lutein/zeaxanthin and α-carotene concentrations, while fruit and juice intakes were positively associated with concentrations of ß-cryptoxanthin. The multivariate models, including dietary variables explained only 15 to 22% of the variance in plasma carotenoid concentrations.

Of the 897 people classified, 46 (5%) were in the top 25% for all plasma carotenoids and classified as the 'hi carotenoid' group. Using the same variables as previously and after excluding 23 people with missing data for hypercholesterolemia or dyslipidemia, being in the hi-carotenoid group was directly associated with being female (versus male), hypercholesterolaemic (versus not hypercholesterolaemic) and SES score (top 20% versus bottom 20%), and inversely associated with smoking (versus not smoking) and abnormal glucose tolerance (IFG/IGT versus normal glucose tolerance) (Table 3). These associations were only slightly modified by the addition of dietary intake data to the model.

**Table 3:** Odds ratios and 95% confidence intervals from multivariate models^a ^looking at factors associated with being in the top 25% for all plasma carotenoid concentrations

Variables	High concentrations for all carotenoids
*Age group (yrs)*	
15-24	1.00
25-34	1.35 (0.47, 3.84)
35-44	1.34 (0.45, 4.00)
45-54	1.98 (0.65, 6.02)
55-64	0.39 (0.04, 3.75)
65+	3.77 (0.55, 25.96)

Sex	
Male	1.00
Female	2.98 (1.24, 7.17)

*SES quintile*	
1	1.00
2	1.93 (0.35, 10.73)
3	2.16 (0.44, 10.47)
4	2.39 (0.45, 12.69)
5	5.58 (1.12, 27.74)
p-trend	0.014

Poor or fair health	1.00
Good or better health	0.62 (0.27, 1.46)

Normal cholesterol	1.00
Hypercholesterolaemia	2.31 (1.14, 4.68)

No dyslipidaemia	1.00
Dyslipidaemia	0.57 (0.26, 1.22)

*Glucose tolerance*	
Normal	1.00
Abnormal^b^	0.08 (0.01, 0.64)
Diabetic	0.29 (0.08, 1.02)

Waist to hip ratio	
Non-obese	1.00
Obese	1.29 (0.54, 3.06)

Body mass index	
Underweight	1.17 (0.34, 4.06)
Normal	1.00
Overweight	1.32 (0.56, 3.12)
Obese	0.70 (0.27, 1.83)

Non-smoker	1.00
Current smoker	0.50 (0.24, 1.06)

Non-alcohol consumer	1.00
Alcohol consumer	1.31 (0.57, 3.00)

^a ^The models included only those variables shown in the table.

^b ^Impaired glucose tolerance and impaired fasting glucose combined.

## Discussion and Conclusions

In this study of urban Indigenous participants, we observed associations between SES and individual plasma carotenoids independent of age, gender, diabetes, lipids, self-rated health, smoking, alcohol use, and obesity. Although intakes of fruit, vegetables and fruit/vegetable juice were positively associated with some plasma carotenoids, and intake of takeaway foods inversely associated with some, adjusting for dietary intake did not completely explain the associations between SES and plasma carotenoids.

**Figure 2:**
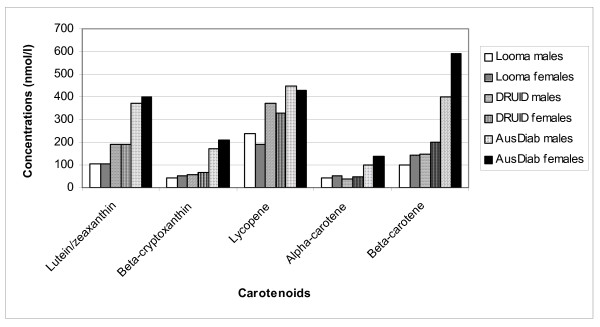
**Mean plasma carotenoid concentration by population**.

In Figure 2 the mean plasma carotenoid concentrations in DRUID are compared with those from two other studies: the Queensland participants of AusDiab [21] and a remote Indigenous community from north western Australia [4]. For all carotenoids, the AusDiab concentrations are highest, with DRUID tending to be between AusDiab and the remote group, although not for α-carotene. Even in the top SES quintile among DRUID participants in Figure 1, the plasma carotenoid concentrations (except lycopene) are lower than the means in the Queensland general population. While this difference could be related to poorer diet quality, it could also relate to the higher rates of smoking and diabetes in the DRUID participants relative to AusDiab [21]. The relatively high lycopene concentrations in the DRUID participants may be attributable to high intakes of processed tomato products in takeaway foods.

As expected, plasma carotenoids, except lycopene, tended to be positively associated with frequency of consumption of fruit, vegetables and fruit/vegetable juice. More frequent intakes of takeaway foods were associated with lower plasma carotenoid concentrations, except for lycopene. These obervations are consistent with our understanding of food sources of carotenoids. Lycopene is typically associated with intake of processed tomato products such as tomato sauce and tomato paste in foods such as pasta and pizza [22,23]. Citrus fruits and orange juice are typically associated with ß-cryptoxanthin, carrots with α- and ß-carotenes; and lutein/zeaxanthin concentrations are associated with consumption of leafy greens [22,23].

The lower circulating carotenoid concentrations in smokers are consistent with other data [24]. Although the high rates of current smoking in DRUID could contribute to low plasma carotenoids, the association with SES was independent of current smoking status. A recent study from Germany found that smoking was associated independently of SES with lower intake of fruit and vegetables [25], which would also impact on plasma carotenoids. An intervention in a remote Aboriginal community aiming to promote the intake of fresh vegetables and fruit was able to improve plasma carotenoids with no change in smoking rates [4]. However, reducing the smoking rate in the DRUID population from the current 40% in both men and women, would likely improve the antioxidant (and general health) status in this group.

After accounting for factors associated with plasma carotenoid concentrations, we observed an association between SES and carotenoids other than βcryptoxanthin. This may be partly attributable to unmeasured confounders; given the relatively small amount of variance explained by our models, there are likely to be other important factors. A US study identified similar factors associated with plasma carotenoids: age, sex, race/ethnicity, carotenoid intake, serum cholesterol, BMI and smoking, explaining between 15% (zeaxanthin) and 26% (ß-carotene) of variance in carotenoids, similar to our results [26]. The lack of explanatory power may also reflect the inaccuracy of the dietary measures or differences in bioavailability of dietary carotenoids across SES classes.

A recent review of diet quality and social class found that groups with a lower socioeconomic status consumed less fruit and vegetables and with less variety than those of higher SES [11]. High energy density diets (ie high in fat and sugar) are associated with lower costs per unit of energy, than high nutrient density diets. In a remote Aboriginal community in northern Australia a direct relationship between dietary quality and food cost was observed [10], with processed foods rich in sugar and fat being much more cost effective sources of energy than fresh fruit and vegetables.

There are other factors beyond cost that may contribute to lower fruit and vegetable intakes in lower SES groups, for example, processed and packaged foods are generally easier to store, last longer, and many require minimal preparation. Exposure to light reduces the levels of carotenoids in fruit and vegetables [27] and limited storage facilities may result in poorer quality fruit and vegetables for lower SES individuals. A recently published survey of health hardware in 132 remote Aboriginal communities found that only 6% of houses had adequate facilities to store, prepare and cook meals [27]. The situation for urban Indigenous participants in the DRUID study is not known, but the possibility exists that inadequate facilities may compromise the nutritional quality of fruit and vegetables consumed by the lower SES groups, as well as limiting the quantity bought. However, the lower than expected concentrations of most carotenoids even in the highest SES quintile suggests that other socio-cultural factors influencing food choice should be investigated.

While the low participation rate in DRUID is a weakness of this study, it is difficult to imagine how this could produce the associations between SES and plasma carotenoids observed. The distribution of participants by age, place of residence, Indigenous group and household income was generally similar to that of the local Indigenous population as measured in the 2001 Census [13]. If participants were of a different SES to the target population, whether lower or higher, although we would expect the latter, this would likely attenuate the observed association by reducing the range of SES included.

As intakes were only recorded at food group levels we were not able to determine the carotenoid contents of the fruits and vegetables consumed. Furthermore, we only have data on the frequency of intake, which does not take into account the amount of foods actually consumed. However, the dietary data did show the expected associations with plasma carotenoids. As explained previously by Cunningham et al [13], there are weaknesses in the reported SES data that may lead to misclassification; however, this is unlikely to be systematic and therefore would be likely to attenuate rather than inflate any associations between SES and carotenoids.

The combined SES score has not been validated; however, it combines variables commonly used in other composite indices of SES [28]. The use of principal components or factor analysis to combine variables in SES indices is not new [29,30], although it has previously been used for area-based indices; and avoids the criticism of some indices that have used arbitrarily selected weights to combine variables [31].

A strength of this study is that it is one of few examining an urban Indigenous population and includes a wide range of SES, from people with no qualifications, a very low income, a large household and who do not own their home, to those with university degrees, in full-time work, a reasonable income and buying or owning their own house. As noted previously, even those classified as high SES in DRUID would not be considered high SES relative to the general Australian population, and would probably be in the middle of the national SES spectrum [13].

In urban Indigenous Australians, higher SES is associated with higher concentrations of plasma carotenoids. This association was not fully explained by dietary intake data. Our data also suggests that even the most well-off among the DRUID participants have lower plasma carotenoids than a sample from the general population in Queensland. Low plasma carotenoids have been linked with poor health outcomes and may explain some of the heavy burden of diabetes and CVD risk observed in the DRUID population [32]. More research is required to identify the significance of low plasma carotenoids in Indigenous Australians, and the most effective way to increase the levels.

## Competing interests

The authors declare that they have no competing interests.

## Authors' contributions

AH analysis plan, data analysis, manuscript preparation. LMB data acquisition, drafting and revising manuscript. JC original study design and data acquisition, drafting and revising manuscript. TD original study design, drafting and revising manuscript. KOD original study design, drafting and revising manuscript. All authors have approved the final manuscript.

## Pre-publication history

The pre-publication history for this paper can be accessed here:

http://www.biomedcentral.com/1471-2458/11/76/prepub
